# Knowledge of Emerging and Reemerging Infectious Diseases in the Public of Guangzhou, Southern China

**DOI:** 10.3389/fpubh.2022.718592

**Published:** 2022-02-08

**Authors:** Xiaowei Ma, Jianyun Lu, Weisi Liu

**Affiliations:** ^1^Department of Public Health Emergency Preparedness and Response, Guangzhou Center for Disease Control and Prevention, Guangzhou, China; ^2^Department of Infectious Disease Control and Prevention, Guangzhou Center for Disease Control and Prevention, Guangzhou, China; ^3^Department of Health Education and Promotion, Guangzhou Center for Disease Control and Prevention, Guangzhou, China

**Keywords:** knowledge, public health, health information, emerging infectious diseases, re-emerging infectious diseases

## Abstract

**Objective:**

The objective of this study is to get the overall picture about the knowledge of emerging and reemerging infectious diseases in public in Guangzhou and provide a scientific basis for developing health information strategies.

**Methods:**

We used the structured questionnaire to interview 1,000 Guangzhou residents by health enquiry hotline. Descriptive analysis was presented to evaluate the knowledge of the participants. Multiple logistic regression model was performed to determine the influence factors for knowledge of emerging and reemerging infectious diseases

**Results:**

A total of 801 individuals completed the survey. About one-third had heard of Middle East respiratory syndrome (MERS) and Zika, whereas Ebola and plague about 50%. A total of 32.08% participants had never heard of any of the four diseases. Only 2.08% knew the sexual transmission of Zika and 90.17% had no idea about the epidemic region of plague. No more than 15% knew they should check their health status after returning from the epidemic region. Education level and income were the key factors that influenced knowledge rate.

**Conclusions:**

The low-level knowledge called for the improvement in health information to the public, especially those with low level of education and income. Effective and precise health information was urged to carry out to improve the prevention for the emerging and reemerging infectious diseases.

## Background

In recent years, emerging and reemerging infectious diseases such as Middle East respiratory syndrome (MERS), Ebola, Zika virus (ZIKV), and plague, have caused great disease burden worldwide. MERS is a highly lethal respiratory disease, and the first case of which was reported in MERS is a highly lethal respiratory disease (1). At the end of November 2019, a total of 2,494 laboratory-confirmed cases and 858 deaths of MERS were reported globally, including Korea and China (2). On May 29, 2015, a 44-year-old male traveler from South Korea to Huizhou, China was confirmed as the first laboratory-confirmed case in China (3), which had caused a panic in the public. Zika virus was first discovered in Uganda in 1947, belonging to mosquito-borne Flavivirus. ZIKV is usually transmitted by the bite of infected mosquitoes. Adults infected with ZIKV are mostly asymptomatic or exhibited mild fever. But for newborn infants, the infection caused severe microcephaly (4, 5). ZIKV is spreading around the world, 89 countries have reported ZIKV cases, and 14 countries in South and Southeast Asia have reported ZIKV infection. Although no Zika local cases were reported in China, a recent study showed that 9.5% (26/273) and 1.8% (5/273) of healthy persons were positive to ZIKV total antibody (IgG and/or IgM) IgM antibody, respectively, in Guangxi Province, which indicated a potential threat to public health (6). Ebola is a rare and often fatal illness caused by Ebolavirus. Infection in human communities is sustained through person-to-person contact. Ebola patients typically experience fever, muscle, pain, fatigue, and headache followed by variable symptoms, such as rash, diarrhea, vomiting, hemorrhagic diathesis, and even cause multiorgan dysfunction (7). From 2014 to 2016, an Ebola outbreak occurred in west Africa, which caused 28,000 cases and 11,325 deaths (7). To date, no confirmed cases of Ebola were reported in China, and China had implemented a serious of effective control and prevention strategy toward Ebola in entry gate, quarantine, national emergency, medical care, personal protection, and environmental sanitation (8). Plague is an acute infectious disease caused by *Yersinia pestis* (*Y. pestis*), which is primarily a disease carried by rodents and is transmitted among animals bitten by infected fleas. Plague killed millions of individuals in Europe in the 14th century. In China, tens of thousands of individuals died of plague in the 19th century (9). In 2017, thousands of plague cases were reported in Madagascar, and about two-third of the cases were pneumonia plague which caused by person-to-person (10). Sporadic plague cases were also reported in Yunnan Province (11) and Beijing city (12) in China in recent year.

Guangzhou, located in Southern China, is the capital city of Guangdong Province and the third largest city in China. As a densely populated urban area with international contacts, Guangzhou has a high risk of imported cases of emerging and reemerging infectious diseases and travel-related illness, although no imported cases of MERS, Ebola, and plague were reported in recent 10 years in Guangzhou. However, there were limited studies about the knowledge of emerging and reemerging diseases in the public in Guangzhou and explore their influencing factor. Hence, our study investigated the level of knowledge of emerging and reemerging infectious diseases, including MERS, Zika, Ebola, and plague among residents of Guangzhou in May 2018, explore the influencing factor to the level of knowledge mentioned above and provided a basis for health information, risk communication, and implementation of measures in an emergency.

## Methods

### Study Participants

Study participants were recruited by telephone interview in Guangzhou residents aged >15 years from a telephone list of 12,320 hotline, which was the official health enquiry hotline in Guangzhou city. Previous studies had used the same method to choose the participants (13, 14).

### Sampling Methods

This cross-sectional survey was conducted from May 7 to May 13, 2018. We calculated the minimum sample size by cross-sectional sampling size estimation methods. The equation was as follows.


$$\begin{array}{l} n=\frac{u_{\partial }^{2}P\left(1-P\right)}{\delta^{2}} \end{array}$$

*n* represents sample size, α is set as 0.05; *P* represents the knowledge rate and was set as 50% in our study to obtain maximum value of P(1−P), and δ is the allowable error and set as 0.05 in our study. The minimum estimation sample size was 385. Due to previous telephone survey study, the response rate is about 60% (13, 15), so the minimum sample size should be 642, and we increase the sample size to 1,000 in our study. The systematic sample method was used to select interviewers in our telephone of 12,320 health enquiry hotline.

### Study Design

A structured questionnaire interview was designed to collect information, divided into basic information and knowledge of emerging and reemerging infectious diseases, including MERS, ZIKA, Ebola, and plague. Basic information included the age, gender, degree of education, degree of income, residential region (suburb or downtown), residence time (less or more than 3 months), and occupation. Knowledge of emerging and reemerging infectious diseases included heard about the diseases, main countries where the outbreak occurred, and preventive ways to diseases. The preventive ways contained that avoiding to the epidemic countries or regions, avoiding to contact with infectious agents such as animals and vectors, and seeking medical care during the incubation period after returning from the epidemic countries or regions if they felt sick.

To ensure the reliability of the questionnaire, three experts for infectious disease were invited to review and revise it. Pilot surveys were conducted prior to the study, to confirm that participants could understand the survey questions and to ensure the validity of the questionnaire content, the Cronbach's coefficient alpha larger than 0.6. The KMO was over 0.6 and the *p-*value of Bartlett's test of sphericity was <0.05. Using the results of this pilot study, the survey questionnaire was amended to create a final version. All questions were either closed-ended or multiple choice.

Investigators contained 10 telephone operators of the Guangzhou 12,320 health enquiry hotline, who underwent training prior to the survey.

### Data Collection and Analysis

EpiData 3.1 (Odense, Denmark) was used to enter data. SPSS 21.0 (Armonk, NY, USA) was used to analyze data. Descriptive analysis was presented to evaluate knowledge of the respondents toward emerging and reemerging infectious diseases. Multiple logistic regression model was performed to determine the influence factors for knowledge of emerging and reemerging infectious diseases (1) MERS, (2) Zika, (3) Ebola, and (4) Plague among respondents. Multiple logistic regression models were used to explain the above four outcomes separately by entering demographics variables of respondents. To assess the level of knowledge, we calculate the score of correct answers for each question of emerging infectious diseases. For some questions with multiple choices, it will have more than one correct option to choose. If the participant chose one correct option, one point will be marked. In other words, if the question has three correct options and the participant got all of them right, he will get three points. Based on the score of answers, the median volume was used as the cutoff point to distinguish “good level of knowledge” (equal to or bigger than the median) from “poor level of knowledge” (less than the median). The good level of knowledge was set as 1, whereas the poor level was set as 0. All variables were entered by forced entry method to compute the adjusted odds ratios (AORs) and the corresponding 95% confidence intervals (CIs). *P*-values which less than 0.05 were considered to be statistically significant for two-sided statistical tests.

### Ethics Approval

The research program and questionnaires were approved by the Ethic Committee of Guangzhou center for disease control and prevention.

Before conducting the survey, we explained the purpose of the survey to the participants and promised to interview them anonymously. The investigation will not begin until obtaining the oral informed consent of the participants. It should be emphasized that if the participants are under 18 years old, the investigators will continue the interview after obtaining their parents or guardians' oral permission.

## Results

### Sociodemographic Characteristics

Among the 1,000 participants who were approached for the interview, a total of 801 agreed to participate and finish the questionnaires, including 337 men (42.07%) and 464 women (57.93%). The effective response rate was 80.1%. A total of 461 respondents were aged 15–35 years (57.55%), and 560 respondents (69.91%) were college or bachelor degree or above. Respondents' income was typically 2,000–10,000 Chinese yuan per month (Table 1).

**Table 1:** Sociodemographic characteristics of the respondents.

**Variable**	***N* (%)**
**Gender**	
Male	337(42.07)
Female	464(57.93)
**Age**	
15~	461(57.55)
35~	294(36.71)
55~	46(5.74)
**Education**	
Senior high/vocational High/secondary technical school and below	241(30.09)
College/bachelor degree or above	560(69.91)
**Income (CY)**	
0~	180(22.47)
5000~	621(77.53)
**Occupation**	
Enterprise staff	366(45.69)
Business and service	131(16.35)
Government organizations	89(11.11)
Unemployed	73(9.11)
Retiree	38(4.74)
Medical staff	34(4.24)
Worker	25(3.12)
Student	22(2.75)
Others	23(2.87)
Blow 3 months	20(2.5)
Upper 3 months	781(97.5)
**Residential area**	
Downtown	547(68.29)
Suburbs	254(31.71)

### Knowledge of Emerging and Reemerging Infectious Diseases

Approximately, a third of the participants had heard of MERS and Zika, whereas half of them had heard of Ebola and plague. A total of 257 individuals (32.08%) had never heard of these diseases. Only 2.08% knew the sexual transmission of Zika and 90.17% had no idea about the epidemic region of plague. No more than 15% knew they should check their health status after returning from the epidemic region (Table 2).

**Table 2:** Related perception of emerging and reemerging infectious diseases.

**Knowledge**	***N* (%)**
**Middle East respiratory syndrome**	
Have you heard of Middle East respiratory syndrome?	221(27.59)
Do you know which countries and regions have respiratory syndrome in the Middle East?	
Unknown	133(60.18)
Choose more than one correct option	88(39.82)
Do you know how to avoid infection with Middle East respiratory syndrome?	
Unknown	159(72.14)
Choose more than one correct option	62(27.86)
**Zika**	
Have you heard of Zika?	240(29.96)
Do you know which countries and regions Zika took place in?	
Unknown	206(85.83)
Choose more than one correct option	34(14.17)
Do you know how to avoid infection with Zika?	
Unknown	177(73.95)
Choose more than one correct option	63(26.05)
**Ebola**	
Have you heard of Ebola?	422(52.68)
Do you know which countries and regions Ebola took place in?	
Unknown	157(37.20)
Choose more than one correct option	265(62.80)
Do you know how to avoid infection with Ebola?	
Unknown	241(57.11)
Choose more than one correct option	181(42.89)
**Plague**	
Have you heard of plague?	407(50.81)
Do you know which countries and regions plague took place in?	
Unknown	383(94.06)
Choose more than one correct option	24(5.94)
Do you know how to avoid infection with plague?	
Unknown	400(98.28)
Choose more than one correct option	7(1.72)

### Logistic Regression Analyses

We used the multiple logistic regression model to analyze the factors associated with the knowledge of participants (Table 3).

**Table 3:** Factors associated with cognitive status of various diseases by multiple logistic regression.

	***N***	***%***	***P***	***OR***	***95%CI***
**MERS**					
Age					
15~	461	57.55		ref	
35~	294	36.71	0.060	1.387	0.986~1.952
55~	46	5.74	0.048	2.094	1.007~4.352
Education level					
Senior high/vocational high/secondary technical school and below	241	30.09		ref	
College/bachelor degree or above	560	69.91	0.000	3.208	2.070~4.973
**Zika**					
Age					
15~	461	57.55		ref	
35~	294	36.71	0.010	1.550	1.112~2.161
55~	46	5.74	0.098	1.848	0.893~3.824
Education level					
Senior High/vocational High/secondary Technical school and below	241	30.09		ref	
College/bachelor degree or above	560	69.91	0.000	2.647	1.759~3.985
Income (CY)					
0~	180	22.47		ref	
5000~	621	77.53	0.005	1.895	1.209~2.970
**Ebola**					
Age					
15~	461	57.55		ref	
35~	294	36.71	0.020	1.458	1.062~2.002
55~	46	5.74	0.080	1.850	0.928~3.687
Education level					
Senior high/vocational high/secondary technical school and below	241	30.09		ref	
College/bachelor degree or above	560	69.91	0.000	2.605	1.842~3.658
Income (CY)					
0~	180	22.47		ref	
5000~	621	77.53	0.001	1.850	1.268~2.698
**Plague**					
Age					
15~	461	57.55		ref	
35~	294	36.71	0.003	1.587	1.169~2.156
55~	46	5.74	0.128	1.658	0.864~3.180
Education level					
Senior high/vocational high/secondary technical school and below	241	30.09		ref	
College/bachelor degree or above	560	69.91	0.000	2.004	1.436~2.798
Income (CY)					
0~	180	22.47		ref	
5000~	621	77.53	0.008	1.636	1.136~2.357

In the model of influence factors of knowledge related to MERS, age 55–65 years (AOR: 2.094, 95% CI: 1.107–4.352) and higher education level (AOR: 3.208, 95% CI: 2.070–4.973) were positively associated with knowledge of participants.

In the models of influencing factors of knowledge related to ZIKA, Ebola, and plague, the positive influencing factors were age 35–54 (AOR: 1.550, 95% CI: 1.112–2.161 for ZIKA; AOR: 1.458, 95% CI: 1.062–2.002 for Ebola, AOR: 1.587, 95% CI: 1.169–2.156 for plague), higher education level (AOR: 2.647, 95% CI: 1.759–3.985 for ZIKA; AOR: 2.605, 95% CI: 1.842–3.658 for Ebola, AOR: 2.004, 95% CI: 1.436–2.798 for plague), and higher monthly income (AOR: 1.895, 95% CI: 1.209–2.970 for ZIKA; AOR:1.850, 95% CI: 1.268–2.698 for Ebola, AOR:1.636, 95% CI: 1.136–2.357 for plague), respectively.

## Discussion

We conducted this survey to provide an overall picture about the public awareness of some emerging and reemerging infectious diseases and explore the influencing factors of knowledge related to MERS, ZIKA, Ebola, and plagues in Guangzhou, Southern China. The effective response rate was 80.1%, which was higher than previous surveys about H7N9 avian influenza in Guangzhou and a study in Italy about travel infectious diseases. This discrepancy hinted that emerging and reemerging infectious diseases draw more and more attention to the public, so they were willing to corporate with our survey. The high response rate might also be attributed to the use of 12,320 official health enquiry hotline, which gave the interviewers a sense of authority and reliability. It implied us to make good use of the official health enquiry hotline to facilitate health information. It was unexpected that <30% participants had heard of MERS and ZIKA, even if the confirmed MERS cases traveled from Korea were reported in Huizhou in 2015, which was closed to Guangzhou city Guangdong province, and the imported ZIKA cases were reported in Guangzhou. A study about Zika virus knowledge in suburban New York city showed that over 90% participants were aware of ZIKA transmission by mosquitoes (16), which was higher than our result about 18.75%. Moreover, the sexual transmission of ZIKA wasseldom mentioned by most of the participants, which was similar to other studies showing low level of knowledge about sexual transmission in Brazil (17), Peru (18), and USA (19). However, the knowledge rates of Ebola and plague were over 50%. The discrepancy might be explained as follows. China government had initiated a national emerging response to Ebola in 2014. Preventive measures including health information were launched to prevent the imported cases of Ebola. Regarding plague, the epidemic of human infection was reported in Guizhou province and Guangxi province in 2000–2002, which was adjacent to the Guangdong Province, and the human plague epidemic and rodent plague epizootic ended in 2005 and 2007, respectively. Then, human plague cases were recently reported in Yunnan Province in 2016 (11). These epidemics caused the attention of the public in Guangzhou city, Guangdong Province. The reasons mentioned above led to a high knowledge rate of Ebola and plague. Although the epidemic of MERS occurred in Middle East (20) and Korea (3), the ZIKA transmission was found in almost all countries in America (21). The public in Guangzhou may thought that they had nothing to do with the epidemic in other countries, so they did not pay enough attention to them. However, <3% knew that they should check their health status after returning from the epidemic area even for the Ebola and plague, which demonstrated a gap in awareness of some of important aspects of emerging and reemerging infectious diseases prevention in the public in Guangzhou. What is more, 32.05% participants have never heard about these four diseases. These findings implied that the local government should facilitate the health information for the emerging and reemerging disease with a risk of imported infection. Proximity to risk is expected to affect an individual's knowledge of the disease and its transmission (22). Health information should focus on preparedness, confidence, counseling, and detailed disaster prevention and mitigation strategies, thereby enhancing the public's sense of social responsibility (23). Media reports of outbreaks are often the main source of the public's knowledge of infectious diseases, but are insufficient for shaping the awareness and helpful health behaviors (24). Therefore, health information disseminated before outbreaks occur played a key role to prevent the emerging infectious diseases (25).

Our findings showed that education and income play the important and positive roles in the knowledge of emerging and reemerging infectious diseases. Although a study about the ZIKA knowledge from suburban New York showed that the education was unrelated to the knowledge (16), our finding was consistent with previous studies in communicable and noncommunicable diseases, which demonstrated that high educational status was correlated with improved vector borne diseases prevention knowledge (19) and the participants with lower educated and those with lower income had higher prevalence of chronic diseases (26). Health inequalities have persisted among different socioeconomic groups (27). It highlighted that we should pay more attention to the public with low education and/or low income on health information. New media and mass media play an increasingly prominent role in health information, but traditional health information activities and counseling are also widespread in Guangzhou. Moreover, health information *via* the Internet should not be the substitute for healthcare professional experts in emerging infectious diseases (15). Taken together, the combination of traditional media, new media, and healthcare professional experts would be a better choice to improve the knowledge about emerging and reemerging infectious diseases in the public.

There were some limitations in our study. First, we used the telephone list from the consultants in the 12,320 health enquiry hotline. The public who consulted the 12,320 hotline would be more trust to the 12,320 hotline, which might cause a bias. Second, we just chose four diseases to represent the emerging and reemerging infectious diseases in our study; however, these diseases had led to epidemic and caused great attention worldwide. Third, although these emerging and reemerging diseases are severe issues in other areas of the world, they did not cause an epidemic in Guangzhou, which would limit the implication of our conclusion.

## Conclusions

We used the telephone survey to get the overall picture about the knowledge of emerging and reemerging infectious diseases in public in Guangzhou. The low-level knowledge of emerging and reemerging infectious disease called for the attention and health information to the public, especially those with low level of education and income. Effective and concise health information was urged to launch to improve the prevention for the emerging and reemerging infectious diseases.

## Data Availability Statement

The original contributions presented in the study are included in the article/supplementary material, further inquiries can be directed to the corresponding author.

## Ethics Statement

The research program and questionnaires were approved by the Ethic Committee of Guangzhou center for disease control and prevention. Before conducting the survey, we explained the purpose of the survey to the participants and promised to interview them anonymously. The investigation will not begin until obtaining the oral informed consent of the participants. It should be emphasized that if the participants are under 18 years old, the investigators will continue the interview after obtaining their parents or guardians' oral permission.

## Author Contributions

WL performed conceptualization and involved in writing, reviewing, and editing. XM and JL performed data curation, involved in methodology and wrote original draft. XM and WL supervised the manuscript and validated the study. JL performed visualization. All authors contributed to the article and approved the submitted version.

## Funding

The study was supported by foundation of science and technology innovation popularization of department of science and technology of Guangdong Province (2019A07076006) and the Medical Health Technology Project for Guangzhou (20201A010045, 20201A011061, and 20201A011062), the Medical Science and Technology Foundation of Guangdong Province (A20211372), and the Key Project of Medicine Discipline of Guangzhou (No.2021–2023-11).

## Conflict of Interest

The authors declare that the research was conducted in the absence of any commercial or financial relationships that could be construed as a potential conflict of interest.

## Publisher's Note

All claims expressed in this article are solely those of the authors and do not necessarily represent those of their affiliated organizations, or those of the publisher, the editors and the reviewers. Any product that may be evaluated in this article, or claim that may be made by its manufacturer, is not guaranteed or endorsed by the publisher.

## Abbreviations

MERS: Middle East respiratory syndrome
ZIKV: Zika virus.
